# Bis(tetra­phenyl­phospho­nium) bis­[*N*-(2,5-dichloro­phenyl­sulfon­yl)dithio­carbimato(2−)-κ^2^ *S*,*S*′]platinate(II)

**DOI:** 10.1107/S1600536810003272

**Published:** 2010-02-03

**Authors:** S. Guilardi, Wilson P. Flauzino Neto, Lucas C. C. Vieira, Raquel S. Amin, Marcelo R. L. Oliveira

**Affiliations:** a Instituto de Química – UFU, 38408-100 Uberlândia, MG, Brazil; b Departamento de Química – UFV, 36571-000 Viçosa, MG, Brazil

## Abstract

In the title salt, (C_24_H_20_P)_2_[Pt(C_7_H_3_Cl_2_NO_2_S_3_)_2_], the Pt^II^ ion (site symmetry 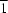) is coordinated by two *S*,*S*′-bidentate *N*-(2,5-dichloro­phenyl­sulfon­yl)dithio­carbimate ligands, resulting in a slightly distorted PtS_4_ square-planar geometry. In the crystal, a C—H⋯O inter­action is observed, as well as electrostatic attraction between the oppositely charged ions.

## Related literature

For other complexes containing a [Pt(*R*SO_2_N=CS_2_)]^2−^ unit, see: Amim *et al.* (2008 ▶); Oliveira *et al.* (2003 ▶, 2004 ▶). For general background to dithio­carbimates, see: Hogarth (2005 ▶). For reference structural data, see: Allen *et al.* (1987 ▶). For further synthetic details, see: Franca *et al.* (2006 ▶).
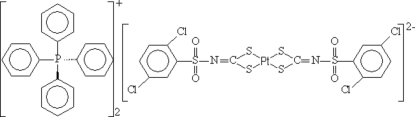


## Experimental

### 

#### Crystal data

(C_24_H_20_P)_2_[Pt(C_7_H_3_Cl_2_NO_2_S_3_)_2_]
*M* *_r_* = 1474.3
Triclinic, 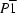

*a* = 9.6284 (1) Å
*b* = 10.3409 (2) Å
*c* = 15.1278 (2) Å
α = 76.951 (1)°
β = 88.353 (1)°
γ = 86.193 (1)°
*V* = 1463.94 (4) Å^3^
*Z* = 1
Mo *K*α radiation
μ = 2.90 mm^−1^
*T* = 120 K
0.34 × 0.34 × 0.3 mm

#### Data collection

Nonius KappaCCD diffractometer
Absorption correction: gaussian (Coppens *et al.*, 1965 ▶) *T*_min_ = 0.439, *T*_max_ = 0.477
11423 measured reflections
6536 independent reflections
6483 reflections with *I* > 2σ(*I*)
*R*_int_ = 0.029

#### Refinement

*R*[*F* ^2^ > 2σ(*F* ^2^)] = 0.025
*wR*(*F* ^2^) = 0.068
*S* = 1.11
6536 reflections
367 parameters
H-atom parameters constrained
Δρ_max_ = 0.69 e Å^−3^
Δρ_min_ = −2.58 e Å^−3^

### 

Data collection: *COLLECT* (Nonius, 2000 ▶); cell refinement: *DENZO* and *SCALEPACK* (Otwinowski & Minor, 1997 ▶); data reduction: *DENZO* and *SCALEPACK*; program(s) used to solve structure: *SHELXS97* (Sheldrick, 2008 ▶); program(s) used to refine structure: *SHELXL97* (Sheldrick, 2008 ▶); molecular graphics: *Mercury* (Macrae *et al.*, 2006 ▶); software used to prepare material for publication: *WinGX* (Farrugia, 1999 ▶).

## Supplementary Material

Crystal structure: contains datablocks global, I. DOI: 10.1107/S1600536810003272/hb5315sup1.cif

Structure factors: contains datablocks I. DOI: 10.1107/S1600536810003272/hb5315Isup2.hkl

Additional supplementary materials:  crystallographic information; 3D view; checkCIF report

## Figures and Tables

**Table:** 

Pt—S1	2.3128 (6)
Pt—S2	2.3233 (6)

**Table:** 

S1—Pt—S2	74.59 (2)

**Table 2:** Hydrogen-bond geometry (Å, °)

*D*—H⋯*A*	*D*—H	H⋯*A*	*D*⋯*A*	*D*—H⋯*A*
C27—H27⋯O1^i^	0.95	2.43	3.111 (4)	128

Symmetry code: (i) 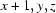.

**Fig. 1.**
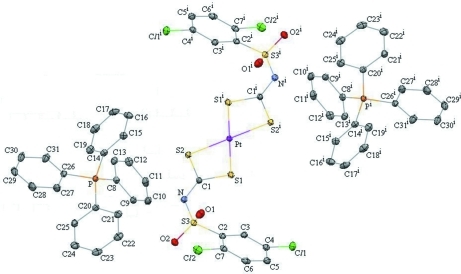
View of (I) with displacement ellipsoids drawn at the 30% probability level and H atoms omitted for clarity.

## supplementary crystallographic information

### Comment

We became interested in the syntheses and characterization of Pt(II) complexes with dithiocarbimates due to their potential application as antitumoral. Some platinum- dithiocarbimato-anionic complexes with general formulae [Pt(RSO_2_N═CS_2_)]^2-^ (R = aryl groups) have had their structures determined by X-ray diffraction techniques. All of these compounds have the tetrabutylammonium as counter-ion (Amim *et al.*, 2008; Oliveira *et al.*, 2004). Variations in the counter-ions and in the R group can be important to modulate the activity of these compounds favoring the biological application.

The title compound is quite stable at the ambient conditions. The Pt(II) is located at the inversion centre and the PtS_4_ fragment has a distorted square-planar geometry due to the bidentate chelation (Figure 1). The Pt—S bond lengths are almost equal but the angles S1—Pt—S2 and S2^i^—Pt—S1 are 74.59 (2)° and 105.41 (2)° respectively (Table 1). In the fragment N═ CS_2_, the C—S bond lengths are nearly equal and are shorter than C—S single bonds (ca 1.815 Å) ( Allen**et al.**, 1987). The C1═N bond distances [1.310 (3) Å] have a double character. This behavior indicates that the electron density is delocalized over the entire NCS_2_ moiety. The S1—C1—N angle is significantly greater than S2—C1—N probably due to the repulsive interaction between the (2,5-Cl_2_C_2_H_3_)SO_2_ group and the S1 atom, which are in *cis* position in relation to the C1—N bond. Similar behavior is observed in the square-planar platinum(II) and nickel(II) complexes of dithiocarbimates (Amim *et al.*, 2008; Oliveira *et al.*, 2004; Oliveira *et al.*, 2003; Franca *et al.*, 2006).

The bond lengths and angles of the tetraphenylphosphonium cations are in agreement with the expected values (Allen *et al.*, 1987). The crystal packing is mainly maintained by ionic bond, but there are weak interactions of the type C—H···O (Table 2).

### Experimental

Potassium 2,5-dichlorophenylsulfonyldithiocarbimate dihydrate was prepared from the sulfonamide using procedures described in the literature (Franca *et al.*, 2006). The title compound was prepared in 1:1 (10 ml) methanol:water mixture from potassium tetrachloroplatinate(II) (0.40 mmol) potassium 2,5-dichlorophenylsulfonyldithiocarbimate dihydrate (0.80 mmol) and tetraphenylphosphonium bromide (0.80 mmol). The reaction mixture was stirred for 1 h at room temperature. The yellow solid obtained was filtered, washed with distilled water, ethyl alcohol and dried under reduced pressure. The title compound is slightly soluble in chloroform and insoluble in water and in most organic solvents.  Yellow prisms of (I) were obtained after slow evaporation of solution of the compound in hot chloroform. M.p. 195.2-195.6°C. IR (most important bands, cm^-1^): 1409 ν(C═N); 1309 ν_ass_(SO_2_); 1107 ν_sym_(SO_2_); 932 ν_ass_(CS_2_) and 312 ν(NiS).

### Refinement

All H atoms were fixed geometrically and allowed to ride on their parent atoms, with C—H distances of 0.95 Å, and with U_iso_(H) = 1.2 U_eq_(C).

### Crystal data

**Table:**

(C_24_H_20_P)_2_[Pt(C_7_H_3_Cl_2_NO_2_S_3_)_2_]	*Z* = 1
*M**_r_* = 1474.3	*F*(000) = 736
Triclinic, *P*1	*D*_x_ = 1.672 Mg m^−^^3^
*a* = 9.6284 (1) Å	Mo *K*α radiation, λ = 0.71073 Å
*b* = 10.3409 (2) Å	Cell parameters from 20612 reflections
*c* = 15.1278 (2) Å	θ = 2.9–27.1°
α = 76.951 (1)°	µ = 2.90 mm^−^^1^
β = 88.353 (1)°	*T* = 120 K
γ = 86.193 (1)°	Prism, yellow
*V* = 1463.94 (4) Å^3^	0.34 × 0.34 × 0.3 mm

### Data collection

**Table:**

Nonius KappaCCD diffractometer	6483 reflections with *I* > 2σ(*I*)
CCD rotation images, thick slices scans	*R*_int_ = 0.029
Absorption correction: gaussian (Coppens *et al.*, 1965)	θ_max_ = 27.3°, θ_min_ = 2.9°
*T*_min_ = 0.439, *T*_max_ = 0.477	*h* = −12→12
11423 measured reflections	*k* = −13→13
6536 independent reflections	*l* = −19→19

### Refinement

**Table:**

Refinement on *F*^2^	0 restraints
Least-squares matrix: full	H-atom parameters constrained
*R*[*F*^2^ > 2σ(*F*^2^)] = 0.025	*w* = 1/[σ^2^(*F*_o_^2^) + (0.0373*P*)^2^ + 0.5896*P*] where *P* = (*F*_o_^2^ + 2*F*_c_^2^)/3
*wR*(*F*^2^) = 0.068	(Δ/σ)_max_ = 0.001
*S* = 1.11	Δρ_max_ = 0.69 e Å^−^^3^
6536 reflections	Δρ_min_ = −2.58 e Å^−^^3^
367 parameters	

### Special details

**Table:**

Geometry. All esds (except the esd in the dihedral angle between two l.s. planes) are estimated using the full covariance matrix. The cell esds are taken into account individually in the estimation of esds in distances, angles and torsion angles; correlations between esds in cell parameters are only used when they are defined by crystal symmetry. An approximate (isotropic) treatment of cell esds is used for estimating esds involving l.s. planes.

### Fractional atomic coordinates and isotropic or equivalent isotropic displacement parameters (Å^2^)

**Table:**

	*x*	*y*	*z*	*U*_iso_*/*U*_eq_	
P	0.48411 (6)	1.00796 (6)	0.28241 (4)	0.01728 (12)	
C13	0.5666 (3)	0.7769 (2)	0.40148 (18)	0.0233 (5)	
H13	0.5906	0.8339	0.4391	0.028*	
C12	0.5927 (3)	0.6407 (3)	0.4298 (2)	0.0271 (5)	
H12	0.6346	0.604	0.4868	0.032*	
C11	0.5571 (3)	0.5583 (3)	0.3744 (2)	0.0287 (6)	
H11	0.5758	0.4649	0.3934	0.034*	
C21	0.3060 (3)	1.0340 (3)	0.13883 (19)	0.0267 (5)	
H21	0.2428	0.9883	0.1824	0.032*	
C10	0.4953 (3)	0.6104 (3)	0.2923 (2)	0.0284 (6)	
H10	0.4709	0.5525	0.2554	0.034*	
C16	0.1901 (3)	1.0494 (3)	0.47488 (19)	0.0299 (6)	
H16	0.144	0.9946	0.5244	0.036*	
C18	0.2237 (3)	1.2660 (3)	0.3820 (2)	0.0323 (6)	
H18	0.2025	1.3593	0.3688	0.039*	
C22	0.2692 (3)	1.0749 (3)	0.0481 (2)	0.0315 (6)	
H22	0.1812	1.0557	0.0291	0.038*	
C17	0.1609 (3)	1.1857 (3)	0.4552 (2)	0.0337 (7)	
H17	0.0971	1.224	0.4925	0.04*	
C25	0.5288 (3)	1.1294 (3)	0.10141 (19)	0.0266 (5)	
H25	0.618	1.147	0.1196	0.032*	
C27	0.7675 (3)	1.0027 (3)	0.2710 (2)	0.0332 (6)	
H27	0.7593	0.9226	0.2513	0.04*	
C24	0.4903 (3)	1.1720 (3)	0.0114 (2)	0.0329 (6)	
H24	0.552	1.2202	−0.0321	0.04*	
C29	0.9089 (4)	1.1685 (4)	0.3024 (2)	0.0442 (8)	
H29	0.9976	1.2034	0.3029	0.053*	
C30	0.7922 (4)	1.2346 (3)	0.3305 (2)	0.0418 (8)	
H30	0.8014	1.3137	0.3513	0.05*	
C28	0.8976 (3)	1.0525 (4)	0.2736 (2)	0.0434 (8)	
H28	0.9785	1.0067	0.2556	0.052*	
C26	0.6493 (3)	1.0705 (3)	0.29743 (17)	0.0228 (5)	
C20	0.4372 (3)	1.0611 (2)	0.16505 (17)	0.0208 (5)	
C14	0.3522 (3)	1.0734 (2)	0.34942 (17)	0.0204 (5)	
C15	0.2866 (3)	0.9923 (3)	0.42239 (18)	0.0237 (5)	
H15	0.3074	0.8989	0.4363	0.028*	
C8	0.5052 (2)	0.8304 (2)	0.31772 (17)	0.0187 (5)	
C9	0.4679 (3)	0.7474 (2)	0.26259 (18)	0.0218 (5)	
H9	0.4248	0.7833	0.2059	0.026*	
C19	0.3180 (3)	1.2109 (3)	0.3275 (2)	0.0274 (5)	
H19	0.3591	1.2659	0.2758	0.033*	
C31	0.6623 (3)	1.1861 (3)	0.3286 (2)	0.0321 (6)	
H31	0.5823	1.2312	0.3483	0.038*	
C23	0.3612 (3)	1.1437 (3)	−0.0145 (2)	0.0332 (6)	
H23	0.3351	1.172	−0.0763	0.04*	
Pt	0	0.5	0.5	0.01621 (5)	
S2	0.17449 (6)	0.63059 (6)	0.42652 (4)	0.02153 (13)	
S3	0.04993 (7)	0.73789 (6)	0.15524 (4)	0.02273 (13)	
S1	−0.07133 (6)	0.57316 (7)	0.35140 (4)	0.02520 (13)	
Cl1	−0.37103 (7)	0.44428 (8)	0.10640 (5)	0.03624 (16)	
Cl2	0.26814 (7)	0.48922 (8)	0.12924 (5)	0.03452 (15)	
C3	−0.1452 (3)	0.5687 (2)	0.13550 (17)	0.0218 (5)	
H3	−0.2096	0.6357	0.1487	0.026*	
C1	0.0838 (2)	0.6508 (2)	0.32596 (17)	0.0190 (5)	
O2	0.1460 (2)	0.7855 (2)	0.08289 (14)	0.0344 (5)	
C2	−0.0029 (3)	0.5823 (2)	0.13914 (16)	0.0200 (5)	
N	0.1355 (2)	0.7139 (2)	0.24836 (15)	0.0231 (4)	
O1	−0.0771 (2)	0.81846 (19)	0.15895 (14)	0.0320 (4)	
C4	−0.1928 (3)	0.4572 (3)	0.11257 (17)	0.0242 (5)	
C7	0.0896 (3)	0.4807 (2)	0.12334 (17)	0.0226 (5)	
C6	0.0405 (3)	0.3690 (3)	0.10070 (18)	0.0281 (6)	
H6	0.1045	0.3001	0.0899	0.034*	
C5	−0.1009 (3)	0.3578 (3)	0.09378 (18)	0.0278 (5)	
H5	−0.1345	0.283	0.0764	0.033*	

### Atomic displacement parameters (Å^2^)

**Table:**

	*U*^11^	*U*^22^	*U*^33^	*U*^12^	*U*^13^	*U*^23^
P	0.0190 (3)	0.0156 (3)	0.0170 (3)	−0.0017 (2)	−0.0019 (2)	−0.0028 (2)
C13	0.0228 (12)	0.0221 (12)	0.0251 (13)	−0.0013 (9)	−0.0040 (10)	−0.0052 (10)
C12	0.0237 (13)	0.0235 (12)	0.0300 (14)	0.0033 (10)	−0.0042 (10)	0.0015 (10)
C11	0.0252 (13)	0.0167 (11)	0.0425 (17)	0.0001 (9)	0.0041 (11)	−0.0039 (11)
C21	0.0263 (13)	0.0263 (12)	0.0263 (14)	0.0009 (10)	−0.0058 (10)	−0.0037 (10)
C10	0.0301 (14)	0.0230 (12)	0.0350 (15)	−0.0046 (10)	0.0033 (11)	−0.0123 (11)
C16	0.0266 (14)	0.0403 (15)	0.0214 (13)	0.0049 (11)	−0.0013 (10)	−0.0059 (11)
C18	0.0323 (15)	0.0271 (13)	0.0409 (17)	0.0102 (11)	−0.0124 (13)	−0.0166 (12)
C22	0.0334 (15)	0.0290 (13)	0.0319 (15)	0.0064 (11)	−0.0140 (12)	−0.0074 (12)
C17	0.0296 (14)	0.0446 (16)	0.0313 (15)	0.0132 (12)	−0.0085 (12)	−0.0215 (13)
C25	0.0354 (14)	0.0208 (12)	0.0235 (13)	−0.0068 (10)	−0.0024 (11)	−0.0028 (10)
C27	0.0246 (14)	0.0462 (17)	0.0323 (15)	−0.0081 (12)	0.0029 (11)	−0.0149 (13)
C24	0.0511 (18)	0.0231 (13)	0.0221 (14)	−0.0038 (12)	−0.0017 (12)	0.0009 (10)
C29	0.0392 (17)	0.065 (2)	0.0252 (15)	−0.0321 (16)	−0.0076 (13)	0.0063 (14)
C30	0.057 (2)	0.0319 (15)	0.0364 (17)	−0.0223 (14)	−0.0183 (15)	0.0017 (13)
C28	0.0235 (14)	0.075 (2)	0.0337 (17)	−0.0164 (15)	0.0035 (12)	−0.0135 (16)
C26	0.0240 (12)	0.0259 (12)	0.0180 (12)	−0.0080 (9)	−0.0040 (9)	−0.0014 (9)
C20	0.0266 (12)	0.0166 (10)	0.0188 (12)	−0.0001 (9)	−0.0047 (9)	−0.0032 (9)
C14	0.0217 (12)	0.0182 (11)	0.0215 (12)	0.0018 (9)	−0.0032 (9)	−0.0055 (9)
C15	0.0241 (12)	0.0234 (12)	0.0228 (12)	0.0026 (9)	−0.0023 (10)	−0.0045 (10)
C8	0.0163 (11)	0.0169 (10)	0.0224 (12)	0.0000 (8)	0.0000 (9)	−0.0037 (9)
C9	0.0212 (12)	0.0234 (12)	0.0218 (12)	−0.0026 (9)	−0.0008 (9)	−0.0069 (10)
C19	0.0307 (14)	0.0204 (12)	0.0302 (14)	0.0016 (10)	−0.0061 (11)	−0.0045 (10)
C31	0.0390 (16)	0.0234 (13)	0.0336 (15)	−0.0049 (11)	−0.0122 (12)	−0.0037 (11)
C23	0.0527 (18)	0.0221 (12)	0.0226 (14)	0.0095 (12)	−0.0118 (13)	−0.0028 (10)
Pt	0.01621 (7)	0.01741 (7)	0.01562 (8)	−0.00152 (4)	0.00092 (5)	−0.00498 (5)
S2	0.0196 (3)	0.0272 (3)	0.0181 (3)	−0.0071 (2)	−0.0012 (2)	−0.0041 (2)
S3	0.0290 (3)	0.0199 (3)	0.0184 (3)	−0.0056 (2)	−0.0033 (2)	−0.0008 (2)
S1	0.0198 (3)	0.0374 (3)	0.0174 (3)	−0.0103 (2)	−0.0013 (2)	−0.0013 (3)
Cl1	0.0297 (3)	0.0462 (4)	0.0361 (4)	−0.0153 (3)	0.0007 (3)	−0.0126 (3)
Cl2	0.0233 (3)	0.0450 (4)	0.0312 (4)	0.0054 (3)	−0.0021 (3)	−0.0021 (3)
C3	0.0247 (12)	0.0227 (11)	0.0175 (12)	−0.0010 (9)	−0.0017 (9)	−0.0032 (9)
C1	0.0185 (11)	0.0182 (11)	0.0209 (12)	−0.0019 (8)	0.0003 (9)	−0.0056 (9)
O2	0.0483 (13)	0.0324 (10)	0.0213 (10)	−0.0184 (9)	0.0020 (9)	0.0011 (8)
C2	0.0257 (12)	0.0193 (11)	0.0134 (11)	−0.0018 (9)	−0.0022 (9)	−0.0001 (9)
N	0.0225 (11)	0.0266 (11)	0.0201 (11)	−0.0069 (8)	−0.0011 (8)	−0.0035 (8)
O1	0.0397 (11)	0.0213 (9)	0.0349 (11)	0.0044 (8)	−0.0116 (9)	−0.0063 (8)
C4	0.0267 (13)	0.0275 (12)	0.0176 (12)	−0.0074 (10)	0.0003 (10)	−0.0016 (10)
C7	0.0235 (12)	0.0250 (12)	0.0161 (11)	0.0012 (9)	−0.0009 (9)	0.0015 (9)
C6	0.0395 (15)	0.0215 (12)	0.0206 (13)	0.0047 (10)	0.0029 (11)	−0.0011 (10)
C5	0.0425 (16)	0.0204 (12)	0.0202 (13)	−0.0068 (10)	0.0014 (11)	−0.0030 (10)

### Geometric parameters (Å, °)

**Table:**

P—C8	1.792 (2)	C29—H29	0.95
P—C14	1.794 (3)	C30—C31	1.382 (4)
P—C26	1.795 (3)	C30—H30	0.95
P—C20	1.797 (3)	C28—H28	0.95
C13—C12	1.384 (4)	C26—C31	1.395 (4)
C13—C8	1.394 (4)	C14—C15	1.390 (4)
C13—H13	0.95	C14—C19	1.404 (3)
C12—C11	1.388 (4)	C15—H15	0.95
C12—H12	0.95	C8—C9	1.394 (3)
C11—C10	1.372 (4)	C9—H9	0.95
C11—H11	0.95	C19—H19	0.95
C21—C22	1.390 (4)	C31—H31	0.95
C21—C20	1.400 (4)	C23—H23	0.95
C21—H21	0.95	Pt—S1^i^	2.3128 (6)
C10—C9	1.396 (4)	Pt—S1	2.3128 (6)
C10—H10	0.95	Pt—S2^i^	2.3233 (6)
C16—C17	1.384 (4)	Pt—S2	2.3233 (6)
C16—C15	1.393 (4)	S2—C1	1.740 (3)
C16—H16	0.95	S3—O2	1.434 (2)
C18—C17	1.376 (5)	S3—O1	1.440 (2)
C18—C19	1.389 (4)	S3—N	1.614 (2)
C18—H18	0.95	S3—C2	1.788 (2)
C22—C23	1.385 (5)	S1—C1	1.735 (2)
C22—H22	0.95	Cl1—C4	1.737 (3)
C17—H17	0.95	Cl2—C7	1.733 (3)
C25—C24	1.386 (4)	C3—C4	1.385 (3)
C25—C20	1.389 (4)	C3—C2	1.391 (4)
C25—H25	0.95	C3—H3	0.95
C27—C28	1.391 (4)	C1—N	1.310 (3)
C27—C26	1.393 (4)	C2—C7	1.390 (3)
C27—H27	0.95	C4—C5	1.385 (4)
C24—C23	1.382 (5)	C7—C6	1.391 (4)
C24—H24	0.95	C6—C5	1.383 (4)
C29—C28	1.377 (5)	C6—H6	0.95
C29—C30	1.383 (6)	C5—H5	0.95
			
C8—P—C14	110.89 (12)	C15—C14—C19	120.1 (2)
C8—P—C26	106.58 (12)	C15—C14—P	121.79 (18)
C14—P—C26	110.24 (12)	C19—C14—P	118.1 (2)
C8—P—C20	111.91 (11)	C14—C15—C16	119.3 (2)
C14—P—C20	108.34 (12)	C14—C15—H15	120.3
C26—P—C20	108.85 (12)	C16—C15—H15	120.3
C12—C13—C8	120.1 (2)	C9—C8—C13	120.4 (2)
C12—C13—H13	120	C9—C8—P	121.96 (19)
C8—C13—H13	120	C13—C8—P	117.59 (18)
C13—C12—C11	119.5 (3)	C8—C9—C10	118.7 (2)
C13—C12—H12	120.3	C8—C9—H9	120.7
C11—C12—H12	120.3	C10—C9—H9	120.7
C10—C11—C12	120.7 (2)	C18—C19—C14	119.5 (3)
C10—C11—H11	119.7	C18—C19—H19	120.2
C12—C11—H11	119.7	C14—C19—H19	120.2
C22—C21—C20	119.1 (3)	C30—C31—C26	119.6 (3)
C22—C21—H21	120.4	C30—C31—H31	120.2
C20—C21—H21	120.4	C26—C31—H31	120.2
C11—C10—C9	120.7 (2)	C24—C23—C22	121.1 (3)
C11—C10—H10	119.6	C24—C23—H23	119.4
C9—C10—H10	119.6	C22—C23—H23	119.4
C17—C16—C15	120.3 (3)	S1^i^—Pt—S1	180
C17—C16—H16	119.8	S1^i^—Pt—S2^i^	74.59 (2)
C15—C16—H16	119.8	S1—Pt—S2^i^	105.41 (2)
C17—C18—C19	120.2 (3)	S1^i^—Pt—S2	105.41 (2)
C17—C18—H18	119.9	S1—Pt—S2	74.59 (2)
C19—C18—H18	119.9	S2^i^—Pt—S2	180.00 (3)
C23—C22—C21	119.9 (3)	C1—S2—Pt	88.44 (8)
C23—C22—H22	120.1	O2—S3—O1	116.81 (13)
C21—C22—H22	120.1	O2—S3—N	106.59 (12)
C18—C17—C16	120.5 (3)	O1—S3—N	111.58 (12)
C18—C17—H17	119.7	O2—S3—C2	106.56 (12)
C16—C17—H17	119.7	O1—S3—C2	105.50 (12)
C24—C25—C20	120.1 (3)	N—S3—C2	109.57 (11)
C24—C25—H25	119.9	C1—S1—Pt	88.90 (9)
C20—C25—H25	119.9	C4—C3—C2	119.8 (2)
C28—C27—C26	119.8 (3)	C4—C3—H3	120.1
C28—C27—H27	120.1	C2—C3—H3	120.1
C26—C27—H27	120.1	N—C1—S1	130.8 (2)
C23—C24—C25	119.4 (3)	N—C1—S2	121.34 (19)
C23—C24—H24	120.3	S1—C1—S2	107.86 (14)
C25—C24—H24	120.3	C7—C2—C3	119.2 (2)
C28—C29—C30	120.6 (3)	C7—C2—S3	123.7 (2)
C28—C29—H29	119.7	C3—C2—S3	116.91 (18)
C30—C29—H29	119.7	C1—N—S3	121.76 (18)
C31—C30—C29	120.3 (3)	C5—C4—C3	121.1 (2)
C31—C30—H30	119.9	C5—C4—Cl1	120.0 (2)
C29—C30—H30	119.9	C3—C4—Cl1	118.9 (2)
C29—C28—C27	119.8 (3)	C2—C7—C6	120.4 (2)
C29—C28—H28	120.1	C2—C7—Cl2	121.7 (2)
C27—C28—H28	120.1	C6—C7—Cl2	117.9 (2)
C27—C26—C31	119.9 (3)	C5—C6—C7	120.4 (2)
C27—C26—P	117.0 (2)	C5—C6—H6	119.8
C31—C26—P	123.0 (2)	C7—C6—H6	119.8
C25—C20—C21	120.4 (2)	C6—C5—C4	119.0 (2)
C25—C20—P	120.7 (2)	C6—C5—H5	120.5
C21—C20—P	118.9 (2)	C4—C5—H5	120.5
			
C8—C13—C12—C11	−0.1 (4)	C20—P—C8—C13	168.17 (19)
C13—C12—C11—C10	−0.6 (4)	C13—C8—C9—C10	−1.0 (4)
C12—C11—C10—C9	0.5 (4)	P—C8—C9—C10	176.1 (2)
C20—C21—C22—C23	−1.2 (4)	C11—C10—C9—C8	0.3 (4)
C19—C18—C17—C16	−0.5 (4)	C17—C18—C19—C14	−2.1 (4)
C15—C16—C17—C18	2.0 (4)	C15—C14—C19—C18	3.3 (4)
C20—C25—C24—C23	−1.2 (4)	P—C14—C19—C18	−175.4 (2)
C28—C29—C30—C31	−1.2 (5)	C29—C30—C31—C26	−0.4 (5)
C30—C29—C28—C27	1.3 (5)	C27—C26—C31—C30	1.8 (4)
C26—C27—C28—C29	0.1 (5)	P—C26—C31—C30	−174.1 (2)
C28—C27—C26—C31	−1.6 (5)	C25—C24—C23—C22	0.7 (4)
C28—C27—C26—P	174.5 (3)	C21—C22—C23—C24	0.5 (4)
C8—P—C26—C27	42.3 (2)	S1^i^—Pt—S2—C1	177.05 (8)
C14—P—C26—C27	162.7 (2)	S1—Pt—S2—C1	−2.95 (8)
C20—P—C26—C27	−78.6 (2)	S2^i^—Pt—S1—C1	−177.05 (8)
C8—P—C26—C31	−141.7 (2)	S2—Pt—S1—C1	2.95 (8)
C14—P—C26—C31	−21.2 (3)	Pt—S1—C1—N	174.9 (2)
C20—P—C26—C31	97.5 (2)	Pt—S1—C1—S2	−4.00 (11)
C24—C25—C20—C21	0.4 (4)	Pt—S2—C1—N	−175.0 (2)
C24—C25—C20—P	−178.8 (2)	Pt—S2—C1—S1	3.98 (11)
C22—C21—C20—C25	0.8 (4)	C4—C3—C2—C7	−2.7 (4)
C22—C21—C20—P	−180.0 (2)	C4—C3—C2—S3	172.41 (19)
C8—P—C20—C25	−112.6 (2)	O2—S3—C2—C7	48.8 (2)
C14—P—C20—C25	124.8 (2)	O1—S3—C2—C7	173.6 (2)
C26—P—C20—C25	4.9 (2)	N—S3—C2—C7	−66.2 (2)
C8—P—C20—C21	68.2 (2)	O2—S3—C2—C3	−126.1 (2)
C14—P—C20—C21	−54.4 (2)	O1—S3—C2—C3	−1.2 (2)
C26—P—C20—C21	−174.3 (2)	N—S3—C2—C3	119.0 (2)
C8—P—C14—C15	4.3 (2)	S1—C1—N—S3	3.5 (3)
C26—P—C14—C15	−113.5 (2)	S2—C1—N—S3	−177.77 (13)
C20—P—C14—C15	127.5 (2)	O2—S3—N—C1	−169.0 (2)
C8—P—C14—C19	−176.98 (19)	O1—S3—N—C1	62.4 (2)
C26—P—C14—C19	65.2 (2)	C2—S3—N—C1	−54.1 (2)
C20—P—C14—C19	−53.8 (2)	C2—C3—C4—C5	0.5 (4)
C19—C14—C15—C16	−1.9 (4)	C2—C3—C4—Cl1	−178.77 (19)
P—C14—C15—C16	176.7 (2)	C3—C2—C7—C6	2.5 (4)
C17—C16—C15—C14	−0.7 (4)	S3—C2—C7—C6	−172.3 (2)
C12—C13—C8—C9	0.9 (4)	C3—C2—C7—Cl2	−178.55 (19)
C12—C13—C8—P	−176.3 (2)	S3—C2—C7—Cl2	6.7 (3)
C14—P—C8—C9	112.1 (2)	C2—C7—C6—C5	−0.1 (4)
C26—P—C8—C9	−127.9 (2)	Cl2—C7—C6—C5	−179.1 (2)
C20—P—C8—C9	−9.0 (2)	C7—C6—C5—C4	−2.1 (4)
C14—P—C8—C13	−70.7 (2)	C3—C4—C5—C6	1.8 (4)
C26—P—C8—C13	49.3 (2)	Cl1—C4—C5—C6	−178.9 (2)

Symmetry codes: (i) −*x*, −*y*+1, −*z*+1.

### Hydrogen-bond geometry (Å, °)

**Table:**

*D*—H···*A*	*D*—H	H···*A*	*D*···*A*	*D*—H···*A*
C27—H27···O1^ii^	0.95	2.43	3.111 (4)	128

Symmetry codes: (ii) *x*+1, *y*, *z*.
